# Fabrication and Adsorption Optimization of Novel Magnetic Core-shell Chitosan/Graphene Oxide/β-cyclodextrin Composite Materials for Bisphenols in Aqueous Solutions

**DOI:** 10.3390/ma13235408

**Published:** 2020-11-27

**Authors:** Yichao Gong, Jianbing Su, Muyuan Li, Aixue Zhu, Guisui Liu, Pengyan Liu

**Affiliations:** 1 College of Chemistry & Environmental Science, Hebei University, No. 180 Wusi East Road, Baoding 071000, China; gyichao@126.com (Y.G.); jianbingsu_cna@163.com (J.S.); Hbulmy@163.com (M.L.); zhuaixue1025@163.com (A.Z.); 13180201082@163.com (G.L.); 2 Key Laboratory of Analytical Science and Technology of Hebei Province, No. 180 Wusi East Road, Baoding 071000, China

**Keywords:** Fe_3_O_4_@SiO_2_/CS/GO/β-CD, adsorption, bisphenol A, bisphenol F, mechanism

## Abstract

A novel magnetic composite material, Fe_3_O_4_@SiO_2_/chitosan/graphene oxide/β-cyclodextrin (MCGC), was prepared by multi-step methods. Various methods were used to systematically characterize the morphology, composition, structure, and magnetic properties of MCGC. The results obtained show that the composite material has good morphology and crystal structure and can be separated quickly by an external magnetic field. The operation is relatively easy, and the raw materials used to prepare this material are economical, easy to obtain, and environmentally friendly. The performance and adsorption mechanism for using this material as an adsorbent to remove bisphenol A (BPA) and bisphenol F (BPF) from water were studied. The adsorption parameters were optimized. Under optimal conditions, MCGC was found to remove more than 90% of BPA and BPF in a mixed solution (20 mg/L, 50 mL); the adsorption process for BPA and BPF on MCGC was found to follow a Redlich–Peterson isotherm model and Pseudo-second-order kinetic model. The adsorption mechanism for MCGC may involve a combination of various forces. Recycling experiments showed that after five uses, MCGC retained a more than 80% removal effect for BPA and BPF, and through real sample verification, MCGC can be used for wastewater treatment. Therefore, MCGC is economical, environmentally friendly, and easy to separate and collect, and has suitable stability and broad application prospects.

## 1. Introduction

Bisphenols are an important type of organic chemical raw material mainly used in the production of polymer materials such as polycarbonate, epoxy resin, and polysulfone resin. They are often added to plastic products to improve stability [1]. However, these bisphenols are also typical endocrine disruptors with estrogen-like effects, and even trace amounts may have adverse effects on human and animal reproductive systems and fetal development. Studies have shown that bisphenol A (BPA) and bisphenol F (BPF) are ubiquitous in various environmental media [2,3]. BPA is currently one of the bisphenol compounds with the largest output and widest application. However, because BPA has an increasingly clear estrogen-like effect, carcinogenicity, mutagenicity, and other hazards, the use of BPA has been restricted in many areas of many countries [4,5]. To meet market demand, substitutes similar to BPA have been gradually developed. As one of the main substitutes for BPA, BPF has been widely used in some household daily necessities, such as food containers, cosmetics, and paper products [6]. Because BPF epoxy resin has lower viscosity and better solvent resistance than BPA epoxy resin, its production has gradually increased in recent years [7]. Studies have reported that BPF is moderately toxic and will induce toxic effects, e.g., reproductive toxicity, cytotoxicity, genetic toxicity, and endocrine interference, after exposure [8]. Through various mechanisms, both BPA and BPF in the environment can enter a water body, allowing them to migrate and transform. Therefore, the establishment of an efficient and rapid method for simultaneously removing BPA and BPF from water has important theoretical and practical significance for reducing the ecological and environmental risks of BPA and BPF.

Chitosan (CS) is the only alkaline natural polysaccharide [9] and is insoluble in water and soluble in acetic acid and most organic acids. It has many amino groups and hydroxyl groups on its surface and many unique properties, including biocompatibility, biodegradability, and nontoxicity, and is renewable and modifiable [10]; thus, CS has good application prospects in adsorption [11], especially for the removal of pollutants in water. To overcome the issues of CS raw materials, including a high degree of crystallization, poor solubility, and difficult recovery [12], commonly used crosslinking agents (glutaraldehyde, glyoxal, etc.) and additional magnetic properties are used to modify CS and improve its stability [13,14]. However, CS modification by crosslinking agents will consume amino groups or hydroxyl groups, resulting in lower adsorption efficiency than that of CS raw materials [12]. Therefore, it is necessary to combine one or more adsorption materials on this basis to compensate. Graphene oxide (GO) is a derivative of graphene (G), and its surface contains many oxygen-containing groups, including carbonyl, hydroxyl, and carboxyl groups, allowing GO to have good dispersibility and to adsorb other compounds via π–π interaction, hydrogen bonding, and hydrophobic interaction [15]. Since the oxygen-containing groups in GO are easily modified, GO and modified GO are often used to adsorb pollutants in water [16,17,18]. *β*-Cyclodextrin (*β*-CD) is a type of cyclodextrin (CD) that has the structural characteristics of a hydrophobic inner cavity and hydrophilic outer surface and can form stable host–guest inclusion complexes with various organic, inorganic, and biological molecules through hydrophobic interactions, electrostatic interactions, van der Waals forces, dipole–dipole interactions, and hydrogen bonding interactions [19,20]. Additionally, *β*-CD is most popular due to its low price and formation of many inclusion complexes. However, CD can easily form intermolecular hydrogen bonds, affecting the adsorption effect, so *β*-CD-modified silica, graphene, and CS are often used as adsorption materials [21,22,23].

In magnetic adsorption composites, Fe_3_O_4_ nanoparticles are often used as the main core structure [24]. The fact that pure Fe_3_O_4_ nanoparticles are easily oxidized in the air can cause problems, such as magnetic property changes, reduced dispersibility, and even loss of active sites on the surface [25]. Currently, one of the most studied modification methods involves coating SiO_2_ onto the surface of Fe_3_O_4_ nanoparticles to improve the oxidation resistance and increase the dispersibility, making the surface easy to modify [26]. Fe_3_O_4_ coated by SiO_2_ can exist stably in acidic solution. A crosslinking method [27] can be used to crosslink CS, the amino group on CS and the carboxyl group on GO to undergo amidation reaction [28], so that Fe_3_O_4_@SiO_2_/CS is grafted onto GO, and, finally, *β*-CD is bonded to the surface of GO through hydrogen bonding. For an adsorbent based on the combination of Fe_3_O_4_@SiO_2_, CS, GO, and *β*-CD, the used raw materials are economical and environmentally friendly, and the preparation, collection and recycling of the adsorbent are relatively easy; furthermore, the number of active sites can be increased and the adsorption capacity can be improved. To our knowledge, there is no research on the application of CS, GO, and *β*-CD functionalized composite magnetic nanoadsorbents in the simultaneous removal of BPA and BPF from water.

In this study, a Fe_3_O_4_@SiO_2_/CS/GO/*β*-CD composite material (MCGC) was successfully prepared and a series of characterizations was carried out. Subsequently, MCGC was used as the adsorbent to optimize the conditions of adsorbent dose, pH, amount of added salt, rotation speed, adsorption temperature, and adsorption time; finally, the adsorption isotherm, adsorption kinetics, and recycling and regeneration performance were evaluated, the adsorption mechanism was discussed, and the material was compared with other adsorption materials. The results show that MCGC has relatively high BPA and BPF removal rates in water, a large adsorption capacity, strong superparamagnetic behavior, and good regenerability, which is convenient for collection, separation, and reuse.

## 2. Materials and Methods

### 2.1. Reagents

Chitosan (CS, ≥90% degree of deacetylation), *β*-cyclodextrin (*β*-CD, 99%), BPF (98%), and glutaraldehyde (50% in water) were purchased from Titan Technology Co., Ltd. (Shanghai, China). GO was purchased from Jining Leader Nano Technology Co., Ltd. (Jining, Shandong, China). BPA (99%) and tetraethyl orthosilicate (TEOS, 98%) were purchased from Mreda Technology Co., Ltd. (Beijing, China). FeCl_3_·6H_2_O (99%), FeSO_4_·7H_2_O (99%), NaCl (99%), acetic acid (99%), and ammonia solution (25%) were purchased from Beichen Fangzhen Chemical Reagent Factory (Tianjin, China). Ethanol (AR grade), acetone (AR grade), and methanol (HPLC grade) were purchased from Kemiou Chemical Reagent Co., Ltd. (Tianjin, China); ultrapure water (prepared by PURELAB Classic, ELGA, High Wycombe, UK) was used in all experiments.

Methanol was used to prepare a 100 mg/L mixed mother liquor of BPA and BPF. Subsequently, water was used for dilution. Since methanol has a cosolvent effect, the volume fraction of methanol should not be higher than 0.001 to eliminate this effect.

### 2.2. Preparation of MCGC

#### 2.2.1. Preparation of Fe_3_O_4_@SiO_2_

Fe_3_O_4_ coated with silica (Fe_3_O_4_@SiO_2_) was prepared by the alkaline coprecipitation method [29] and the sol-gel method. FeCl_3_·6H_2_O and FeSO_4_·7H_2_O were dissolved in water at a molar ratio of 2:1, and the mixture was stirred continuously under a nitrogen environment, followed by the slow addition of aqueous ammonia until the pH reached approximately 10. Subsequently, the mixture was heated at 80 °C for 30 min, and a magnet was then used to collect the Fe_3_O_4_ particles, which were washed with water and ethanol several times. Finally, the Fe_3_O_4_ particles were vacuum dried at 50 °C for 12 h. Then, 0.5 g of Fe_3_O_4_ nanoparticles was added to a mixed solution of 40 mL of water and 160 mL of ethanol, and the mixture was stirred and sonicated for 30 min to uniformly disperse the constituents. Next, 0.5 mL of TEOS and 4 mL of aqueous ammonia were slowly added into the solution dropwise. After the mixture was stirred and mixed to allow the reaction to proceed at 30 °C for 6 h, the solid–liquid was separated with a magnet, and the obtained solid was washed three times with absolute ethanol followed by vacuum drying at 50 °C for 12 h.

#### 2.2.2. Preparation of Fe_3_O_4_@SiO_2_/CS/GO

First, 1 g of CS was dissolved into 100 mL of acetic acid solution (2% v/v), and the mixture was sonicated for 5 min. Subsequently, 0.5 g of Fe_3_O_4_@SiO_2_ was added into the fully dissolved CS solution, and the mixture was stirred vigorously at 30 °C for 2 h. Then, 1 g of GO, 5 mL of glutaraldehyde, and 4 mL of aqueous ammonia were added into the mixed solution, which was stirred at 80 °C for 2 h to allow the crosslinking and amidation reactions to proceed. The obtained solid was collected with a magnet, washed several times with water and ethanol, then dried under vacuum at 50 °C for 12 h.

#### 2.2.3. Preparation of Fe_3_O_4_@SiO_2_/CS/GO/β-CD

**Scheme 1:**
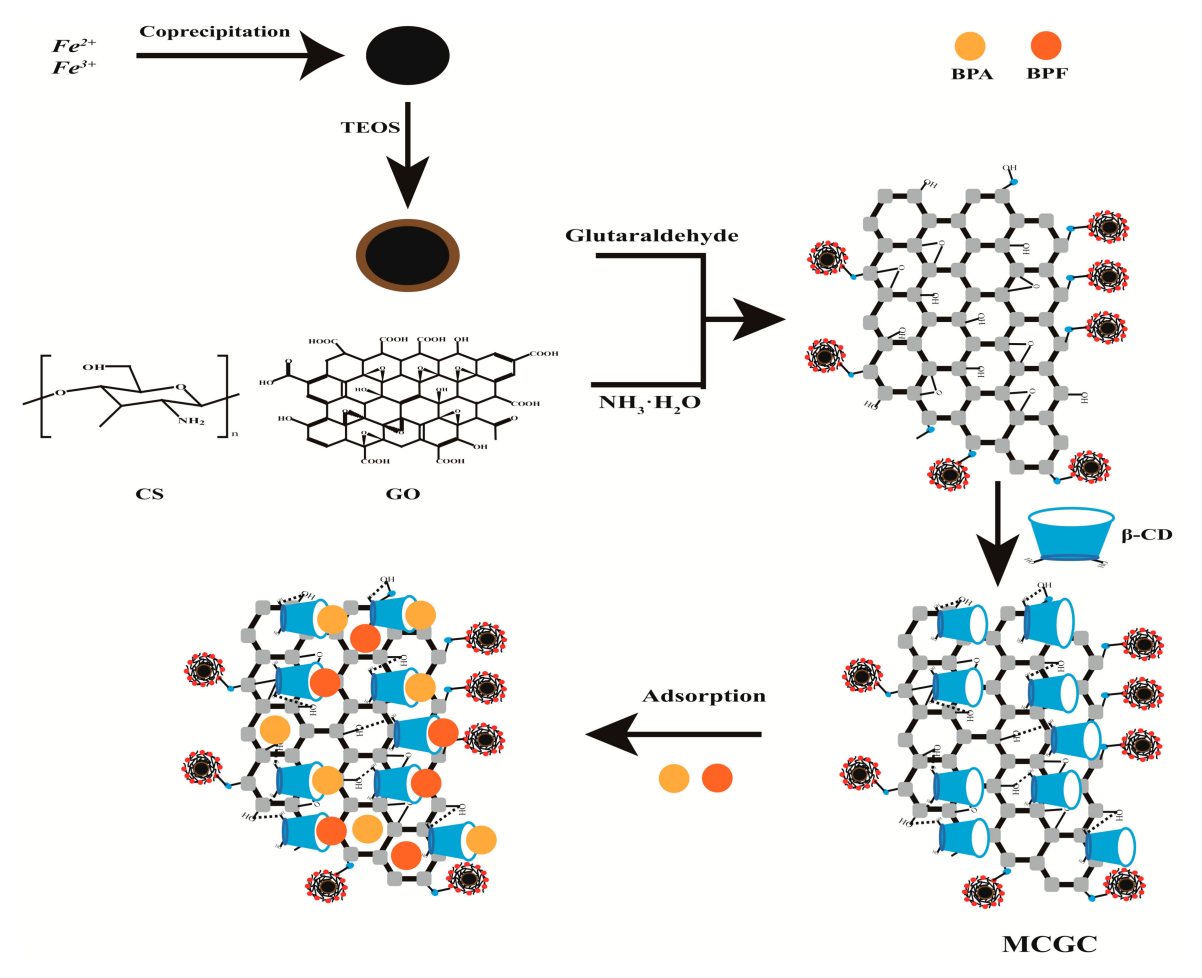
Fabrication process of the novel magnetic composite material, Fe_3_O_4_@SiO_2_/CS/GO/*β*-CD (MCGC).

First, 0.5 g of Fe_3_O_4_@SiO_2_/CS/GO and 5 g of *β*-CD were added to 120 mL of water, and the mixture was sonicated, stirred vigorously for 10 min, then stirred at 60 °C for 5 h. Next, the product was separated and collected with a magnet, washed several times with water, then vacuum dried at 50 °C for 12 h. The final obtained product was MCGC, as shown in Scheme 1.

### 2.3. Characterization

The morphology of the composite material was investigated using scanning electron microscopy (SEM) (QUANTA430, FEI, Hillsboro, OR, USA). The morphology of the composite material was investigated by transmission electron microscopy (TEM) (TECNAI G2 20, FEI, Hillsboro, OR, USA). Fourier transform infrared spectroscopy (FTIR) (NICOLET 5700, Thermo Electron, Waltham, MA, USA) was utilized to identify the surface functional groups using KBr pelleted samples. X-ray diffraction (XRD) patterns were obtained using an XRD analyzer (D8 ADVANCE, Bruker, Germany) utilizing Cu radiation at 40 kV and 40 mA. The hysteresis curve was obtained at room temperature by using a magnetic property measurement system (MPMS SQUID XL, Quantum Design, San Diego, CA, USA).

### 2.4. Adsorption Study

To optimize the conditions for BPA and BPF adsorption on MCGC, an experimental scheme was adopted for batch adsorption of a BPA and BPF mixed solution (initial concentration and volume of 20 mg/L and 50 mL, respectively), and each adsorption experiment was repeated three times. The entire adsorption process was carried out in a Huanida HZ-9211KC open-air constant-temperature shaker (Taicang, China); the pH was adjusted to a fixed value with 0.1 M HCl and NaOH, and the measurement was then carried out with a REX PXSJ-216F ion meter (Shanghai, China). After adsorption was completed, the adsorbent was separated from the solution with a magnet, and the BPA and BPF concentrations remaining in the supernatant were measured using a high-performance liquid chromatograph equipped with a fluorescence detector (HPLC-FLD, Shimadzu, Kyoto, Japan). The measurement conditions were as follows: fluorescence excitation wavelength, 227 nm; emission wavelength, 313 nm; mobile phase, methanol, and water (70:30, v/v); flow rate, 0.8 mL/min; injection volume, 10 μL. Subsequently, the removal rate (*R*) and adsorption capacity (*q_e_*) were calculated according to Equations (1) and (2):

(1) $$\mathrm{Removal}\;\mathrm{rate}:\;R\%=\left(\frac{C_{0}-C_{f}}{C_{0}}\right)\times 100$$

(2) $$\mathrm{Adsorption}\;\mathrm{capacity}:q_{e}=\frac{\left(C_{0}-C_{f}\right)V}{m}$$

where *C*_0_ (mg/L) and *C_f_* (mg/L) represent the initial and final concentrations of BPA and BPF in the solution, respectively, *q_e_* represents the adsorption capacity (mg/g), *V* (L) represents the volume of the adsorption solution, and m (g) represents the mass of the adsorbent.

### 2.5. Adsorption-Desorption Study

After 20 mg of MCGC was added to 50 mL of 20 mg/L BPA and BPF mixed solution, the mixture was shaken at 30 °C and 200 rpm for 1 h, after which the solid and liquid were separated with a magnet and the supernatant was collected and passed through a 0.22 μm nylon membrane for HPLC-FLD analysis. Then, 5 mL of acetone was added to the separated MCGC composite, and the mixture was shaken at 30 °C and 300 rpm for 2 h, after which the solid and liquid were separated with a magnet. The solution was dried by blowing nitrogen at 60 °C, and the residue was dissolved with 1 mL of methanol and passed through a 0.22 μm nylon membrane for HPLC-FLD analysis. The desorption process was repeated three times, and the samples were measured to ensure complete desorption. The separated MCGC was washed several times with water and then dried under vacuum at 50 °C for 3 h. Then, the previous adsorption experiment was repeated, and a total of five adsorption–desorption experiments was carried out.

## 3. Results and Discussion

### 3.1. Characterization

**Figure 1:**
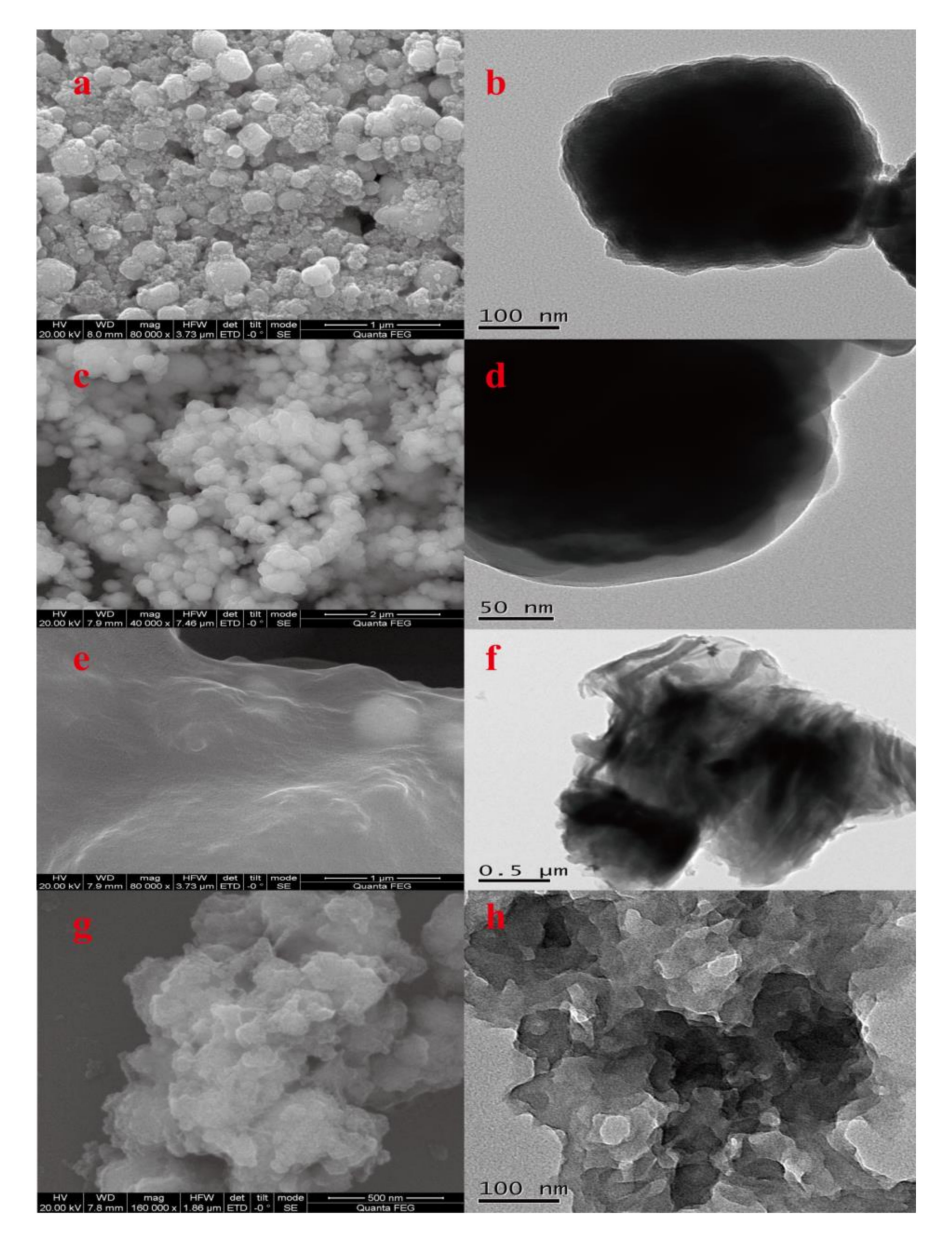
SEM and TEM images of Fe_3_O_4_ (**a**,**b**), Fe_3_O_4_@SiO_2_ (**c**,**d**), Fe_3_O_4_@SiO_2_/CS/GO (**e**,**f**), and Fe_3_O_4_@SiO_2_/CS/GO/*β*-CD (**g**,**h**).

Figure 1 shows SEM and TEM images of Fe_3_O_4_, Fe_3_O_4_@SiO_2_, Fe_3_O_4_@SiO_2_/CS/GO, and Fe_3_O_4_@SiO_2_/CS/GO/*β*-CD; the SEM images are on the left-hand side (a, c, e, and g), while the TEM images are on the right-hand side (b, d, f, and h).

As shown in Figure 1a,b, the synthesized Fe_3_O_4_ was approximately spherical, and some particles were agglomerated, with a diameter of approximately 200–250 nm. Figure 1c,d show that the thickness of Fe_3_O_4_@SiO_2_ increased by approximately 5–10 nm; the surface became smooth, and the agglomeration phenomenon was considerably reduced. This result occurred because the SiO_2_ coating not only reduces the dipole–dipole interaction between particles to prevent oxidation and reduce aggregation but also improves dispersibility and stability, which is beneficial for modification. Figure 1e,f indicate that the surface of the material is wrinkled, with a large flake-like structure visible. In addition, there were wrinkled flake-like structures on the edge, indicating that GO was successfully combined with Fe_3_O_4_@SiO_2_/CS and that the diameter of Fe_3_O_4_@SiO_2_/CS increased to 400–500 nm. As shown in Figure 1g,h, the surface of Fe_3_O_4_@SiO_2_/CS/GO/*β*-CD was rougher than that found for the previously synthesized intermediate. Figure 1h indicates distinct *β*-CD accumulation on the surface of Fe_3_O_4_@SiO_2_/CS/GO.

**Figure 2:**
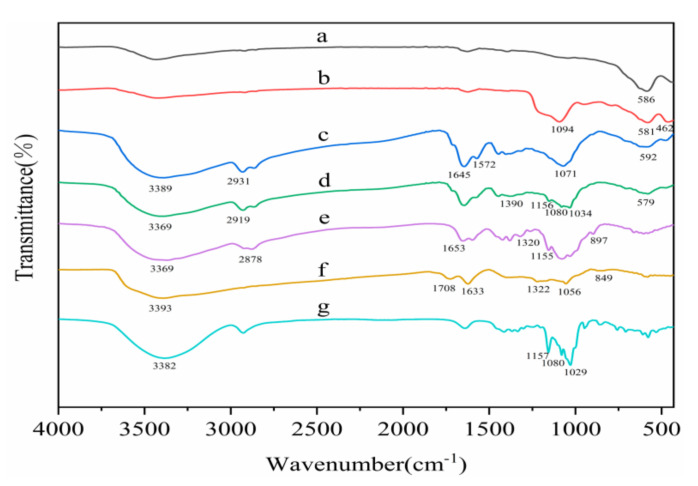
FTIR spectra of Fe_3_O_4_ (**a**), Fe_3_O_4_@SiO_2_ (**b**), Fe_3_O_4_@SiO_2_/CS (**c**), Fe_3_O_4_@SiO_2_/CS/GO/*β*-CD (**d**), CS (**e**), GO (**f**), and *β*-CD (**g**).

The surface groups on MCGC were studied by FTIR, and the results are shown in Figure 2. Curves a, b, c, and d show the IR spectra obtained for Fe_3_O_4_, Fe_3_O_4_@SiO_2_, Fe_3_O_4_@SiO_2_/CS/GO and Fe_3_O_4_@SiO_2_/CS/GO/*β*-CD, respectively, and the IR spectra of curves e (CS), f (GO), and g (*β*-CD) are shown for reference.

The peak at 586 cm^−1^ in curve a corresponds to the Fe–O–Fe stretching vibration; the peaks at 1094 cm^−1^ and 462 cm^−1^ in curve b are associated with Si–O–Si asymmetric stretching vibration and Si-O stretching vibration, respectively, indicating that SiO_2_ was successfully coated onto the Fe_3_O_4_ surface. The peaks observed between 897 cm^−1^ and 1155 cm^−1^ in curve e are due to the C–O–C stretching vibration and the C–O stretching vibration, respectively, and the peak at 1320 cm^−1^ reflects the C–N stretching vibration. In addition, the peak at 3369 cm^−1^ is due to the O–H stretching vibration, and the peaks at 1653 cm^−1^ and 2878 cm^−1^ indicate the presence of N–H bonds. The peak at 1633 cm^−1^ in curve f is assigned to the C=C stretching vibration, the peak at 1708 cm^−1^ is due to the C=O stretching vibration, and the peaks between 849 cm^−1^ and 1322 cm^−1^ are associated with the stretching vibration peak of the epoxy group. In curve c, the same functional groups can be found around the above wavenumbers, but due to the amidation reaction between the amino group of CS and the carboxyl group of GO, the C=O stretching vibration peak at 1708 cm^−1^ disappeared, and the characteristic peaks for the NH mixed vibration and C=O stretching vibration appeared at 1572 cm^−1^ and 1645 cm^−1^, respectively. Compared with that in curve c, the area of the OH bending vibration peak in curve d at 1390 cm^−1^ in curve d was greater, which is attributed to the presence of a C-H/O-H bending vibration peak, a coupled C–O/C–C bending vibration peak, and an OH stretching vibration peak. Typical characteristic peaks were observed at 1034 cm^−1^, 1080 cm^−1^, and 1156 cm^−1^ in curve g, and the O–H stretching vibration peak at 3369 cm^−1^, the corresponding vibration peak (3389 cm^−1^) for Fe_3_O_4_@SiO_2_/CS/GO, and the free OH vibration peak (3700 cm^−1^) redshifted. These findings indicate that MCGC was successfully prepared and that a strong hydrogen bonding interaction exists between *β*-CD and Fe_3_O_4_@SiO_2_/CS/GO.

**Figure 3:**
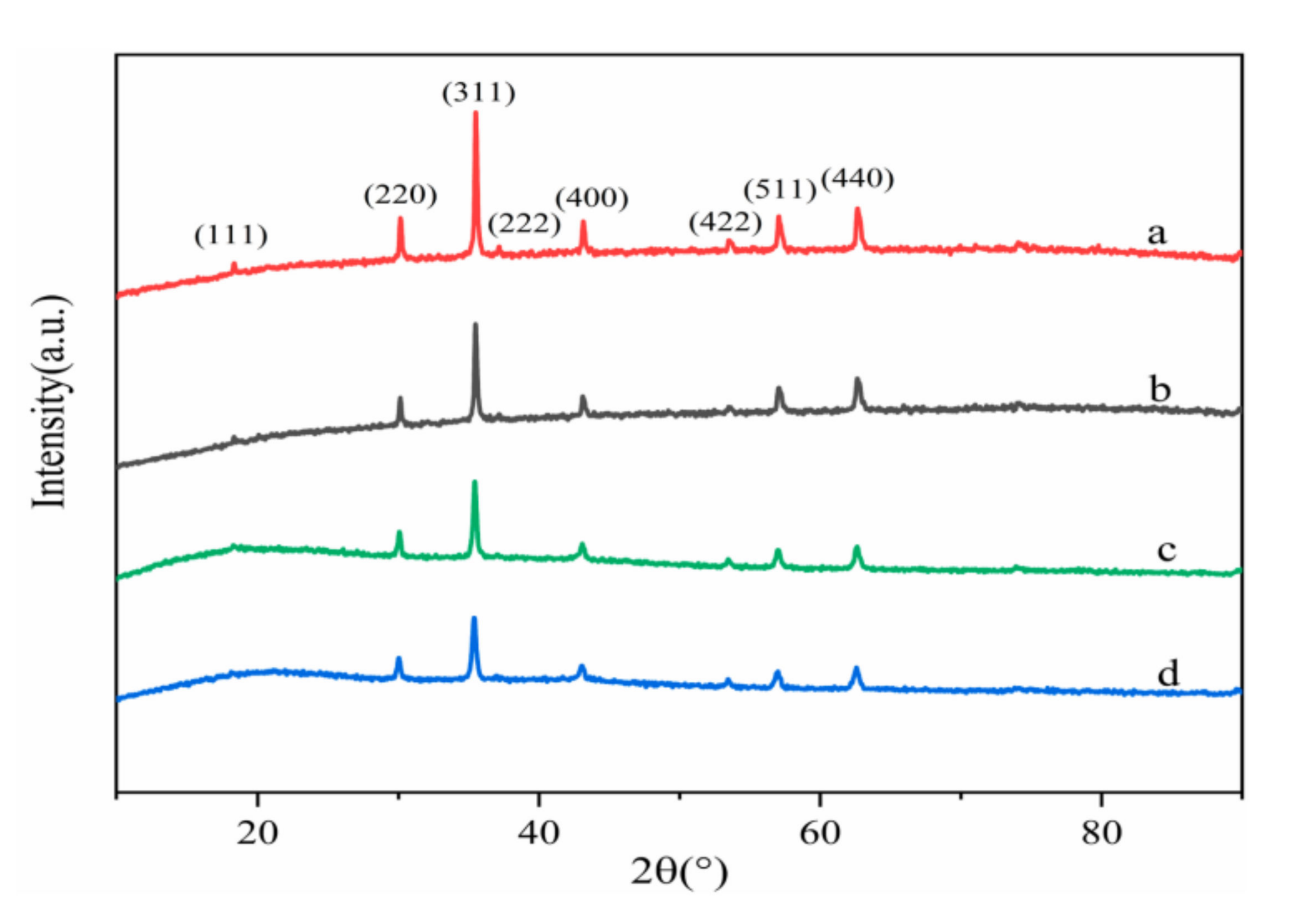
XRD patterns of Fe_3_O_4_ (**a**), Fe_3_O_4_@SiO_2_ (**b**), Fe_3_O_4_@SiO_2_/CS (**c**), and Fe_3_O_4_@SiO_2_/CS/GO/*β*-CD (**d**).

The XRD patterns obtained for Fe_3_O_4_ (a), Fe_3_O_4_@SiO_2_ (b), Fe_3_O_4_@SiO_2_/CS/GO (c), and Fe_3_O_4_@SiO_2_/CS/GO/*β*-CD (d) are shown in Figure 3.

The six main characteristic peaks due to Fe_3_O_4_ were observed in all materials, i.e., 30.128° (220), 35.544° (311), 43.157° (400), 53.55° (422), 57.063° (511), and 62.699° (440), which is consistent with the standard structure of an Fe_3_O_4_ crystal (JCPDS card no. 89-0950), suggesting that no loss of Fe_3_O_4_ occurred during MCGC preparation. However, due to the introduction of CS, GO, and *β*-CD, the characteristic peak intensity due to Fe_3_O_4_ decreased, indicating that the Fe_3_O_4_ content in MCGC decreased.

**Figure 4:**
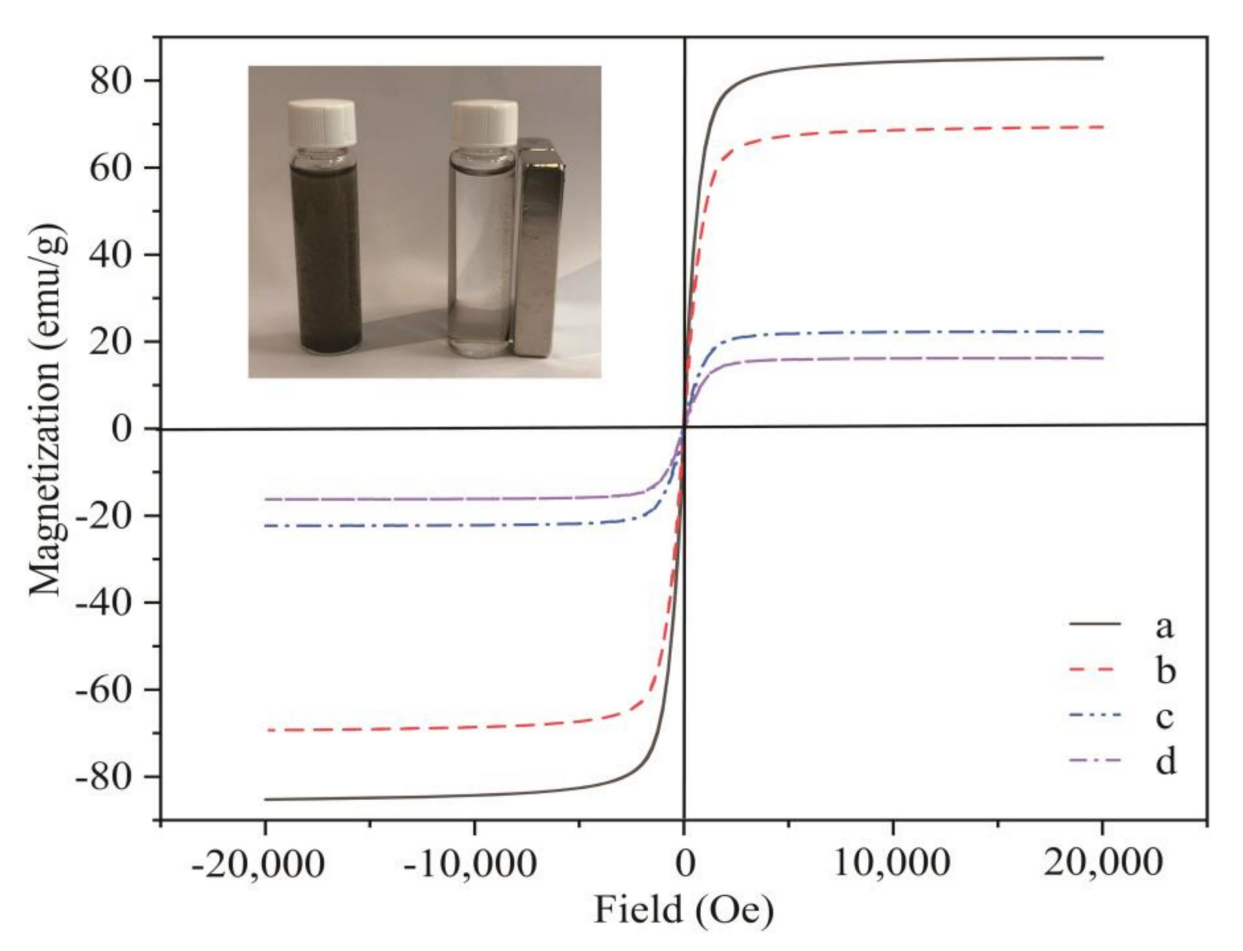
The magnetic hysteresis of Fe_3_O_4_ (**a**), Fe_3_O_4_@SiO_2_ (**b**), Fe_3_O_4_@SiO_2_/CS/GO (**c**), and Fe_3_O_4_@SiO_2_/CS/GO/*β*-CD (**d**).

A magnetic measurement system was used to study the magnetic properties of the materials at room temperature. Figure 4 shows the magnetic hysteresis curves obtained for Fe_3_O_4_ (a), Fe_3_O_4_@SiO_2_ (b), Fe_3_O_4_@SiO_2_/CS/GO (c), and Fe_3_O_4_@SiO_2_/CS/GO/β-CD (d), with maximum saturation magnetization values of 85.3 emu/g, 69.3 emu/g, 22.3 emu/g, and 16.2 emu/g, respectively.

The magnetization of MCGC was found to gradually decrease after the introduction of CS, GO, and *β*-CD compared with that of Fe_3_O_4_ and Fe_3_O_4_@SiO_2_. Figure 4 indicates that even though the MCGC magnetization decreased, its residual magnetic force was sufficient for separation via an applied magnetic field. The above results show that after MCGC adsorbs BPA and BPF in water, it can be separated within seconds by applying a magnetic field.

### 3.2. Study to Optimize Adsorption

#### 3.2.1. Optimization of the Adsorbent Dosage

**Figure 5:**
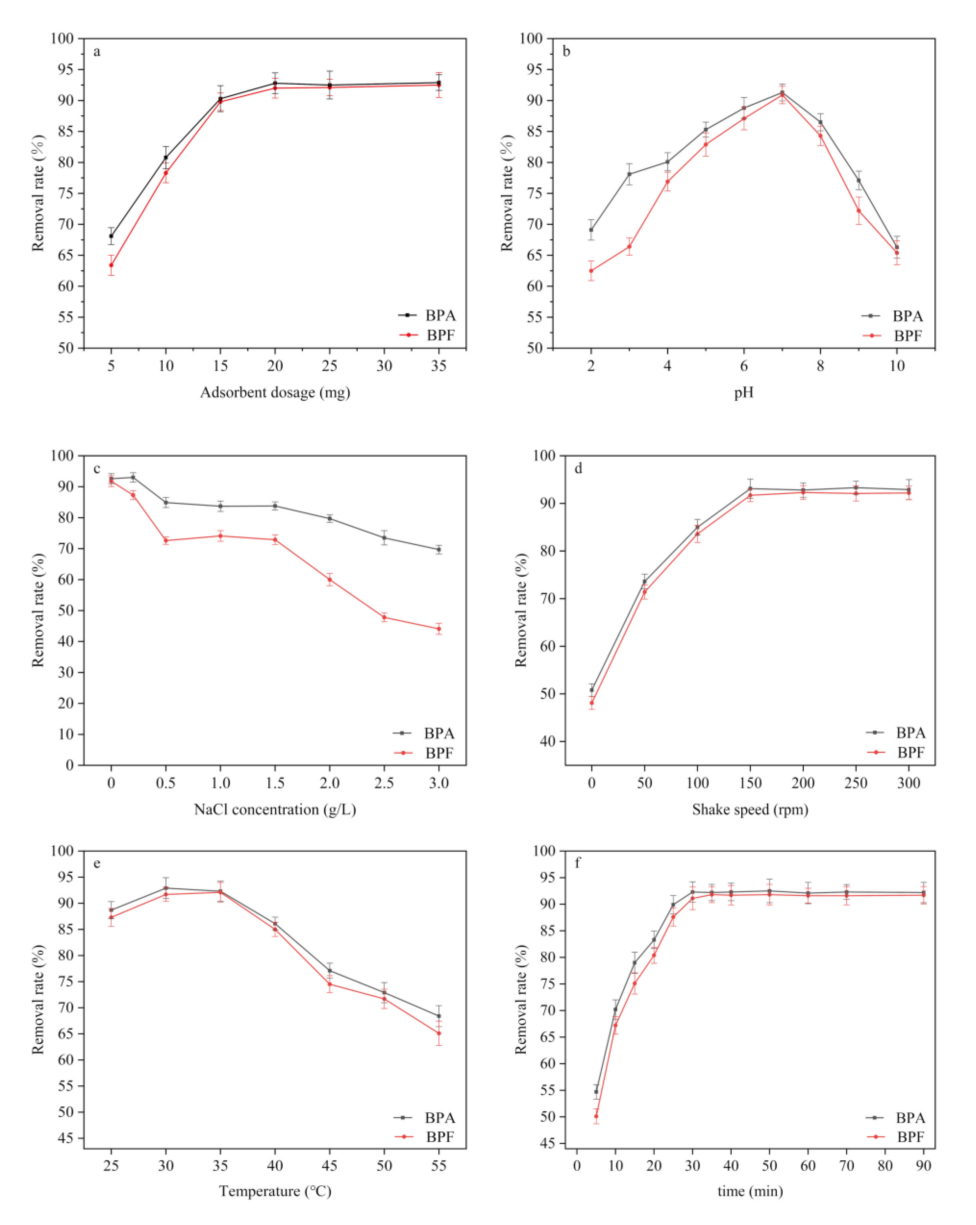
Optimization study for the use of MCGC to remove bisphenol A (BPA) and bisphenol F (BPF): adsorbent dosage (**a**); pH (**b**); ionic strength (**c**); shaking speed (**d**); temperature (**e**); time (**f**).

The adsorbent dosage is an important condition affecting adsorbent performance [30]. At an initial concentration of 20 mg/L for the BPA and BPF mixed solution, volume of 50 mL, solution pH of 7, adsorption temperature of 30 °C, shaking speed of 200 rpm, and adsorbent dosage in the range of 5–35 mg, the impact of MCGC on the BPA and BPF adsorption capacities was studied. MCGC adsorbs BPA and BPF, mainly due to π–π interaction, hydrophobic interaction, and the formation of host-guest inclusion complexes [31,32]. Figure 5a shows that the BPA removal rate was slightly higher than the BPF removal rate, which may be because BPF is slightly more polar due to the presence of two methyl groups on the central carbon atom that are absent in BPA [33].

Additionally, with increasing adsorbent dosage, the BPA and BPF adsorption capacities increased rapidly. The adsorption capacity reached its maximum with an adsorbent dosage of 20 mg, and the BPA and BPF removal rates were approximately 92.8% and 92%, respectively. A continuous increase in the adsorbent dosage did not lead to a great change in the adsorption capacity. Therefore, an adsorbent dosage of 20 mg was used for the following experiments.

#### 3.2.2. Optimization of pH

The pH value can affect the active sites on the adsorbent and the existing form of the adsorbate, thus affecting the adsorption performance of the adsorbent [34,35]. With the other adsorption conditions remaining unchanged, the effect of the adsorbent on the BPA and BPF adsorption capacities was studied by changing the pH value (2–10). Figure 5b shows that for a pH of 7, MCGC exhibited the largest BPA and BPF removal rates. The BPA and BPF removal rates gradually increased as the pH was increased from 2 to 7 and gradually decreased as the pH was further increased to 10. BPA and BPF are both bisphenols, and the pKa values of the two phenolic hydroxyl groups range between 9.6 and 10.2 [36]. When the solution is acidic, although both BPA and BPF are in a molecular state, much of the H^+^ and H_3_O^+^ ions in the solution may occupy the adsorption sites on the adsorbent, so that the number of adsorption sites for adsorbing BPA and BPF is reduced. Furthermore, the epoxy groups on the adsorbent are also charged under acidic conditions, so that some adsorption sites may be occupied by more water molecules, reducing the adsorption of other compounds with relatively weak polarity. When the solution is alkaline, the functional groups, e.g., carboxyl and hydroxyl groups, on the surface of the adsorbent are negatively charged, and both BPA and BPF are also ionized to produce bisphenol anions. The adsorbent, BPA, and BPF are all negatively charged, although the adsorbent can undergo anion–π interactions with BPA and BPF, and the adsorption capacity may decrease due to electrostatic repulsions. Therefore, for MCGC to adsorb BPA and BPF, the optimum pH was 7.

#### 3.2.3. Optimization of Ionic Strength

Adding inorganic salts to the solution may lead to changes in the hydrophobicity, surface charge, and solubility of the adsorbate, which in turn affects the adsorption performance of the adsorbent [37]. Under the same conditions, the effect on adsorption capacity was studied by adding 0-3 g/L NaCl to the solution. The result is shown in Figure 5c. When the amount of salt added ranged from 0 g/L to 0.2 g/L, the BPF removal rate gradually decreased and the BPA removal rate slightly increased, although this change was not significant. This result may have occurred because a low ionic strength can slightly increase the hydrophobicity of BPA with only a minor effect on BPF, which increases the number of active sites for adsorbing BPA on the adsorbent and thus results in a decrease in the BPF adsorption capacity. When the amount of added salt was increased, the BPA and BPF removal rates were significantly reduced. It is possible that the density of the solution increases due to the addition of large quantities of inorganic salts, which increases the diffusion resistance of BPA and BPF to the adsorbent. Therefore, to ensure maximum adsorption of BPA and BPF, no salts were added in subsequent experiments.

#### 3.2.4. Optimization of Shaking Speed

The shaking speed can affect the contact between the adsorbent and the adsorbate, thereby affecting the adsorption capacity of the adsorbent [38]. Figure 5d shows that as the shaking speed was increased from 0 rpm to 150 rpm, the BPA and BPF removal rates gradually increased. The maximum BPA and BPF removal rates were obtained with a shaking speed of 150 rpm. A continuous increase in the shaking speed was found to have a minor effect on the BPA and BPF removal rates. This finding shows that a moderate increase in the shaking speed can increase the BPA and BPF diffusion rates to the adsorbent. Therefore, the optimal shaking speed was determined to be 150 rpm.

#### 3.2.5. Optimization of the Adsorption Temperature

To analyze the influence of temperature on BPA and BPF removal, an adsorption experiment was carried out by changing the temperature (25–55 °C) with the other adsorption parameters remaining unchanged. Figure 5e shows that as the temperature was increased from 25 °C to 30 °C, the BPA and BPF removal rates also increased, possibly because an increase in temperature is beneficial to the diffusion of BPA and BPF to the adsorbent. As the temperature was increased from 30 °C to 35 °C, the BPF removal rate remained essentially unchanged, while the BPA removal rate decreased slightly. Upon further increasing the temperature, the BPA and BPF removal rates gradually decreased, indicating that this adsorption process may be exothermic and that an increase in the temperature is not conducive to adsorbent adsorption. Therefore, 30 °C was selected as the optimum adsorption temperature.

#### 3.2.6. Optimization of Adsorption Time

The previously optimized adsorption conditions were used to study the effect of adsorption time within the time range of 5–90 min. Figure 5f shows the changes in the BPA and BPF removal rates over time. Figure 5f indicates that as time increased, the BPA and BPF removal rates both increased rapidly. The maximum BPA and BPF removal rates occurred at adsorption times of 30 and 35 min, respectively. A continuous increase in the adsorption time had a minor effect on the BPA and BPF removal rates. This result may be due to BPF having two fewer methyl groups than BPA, resulting in slightly less hydrophobicity and thus providing BPA a slight advantage when competing for the same active site. Therefore, because the equilibrium time for BPF adsorption was slightly longer than that for BPA, 35 min was selected as the optimal adsorption time.

### 3.3. Adsorption Isotherm

**Figure 6:**
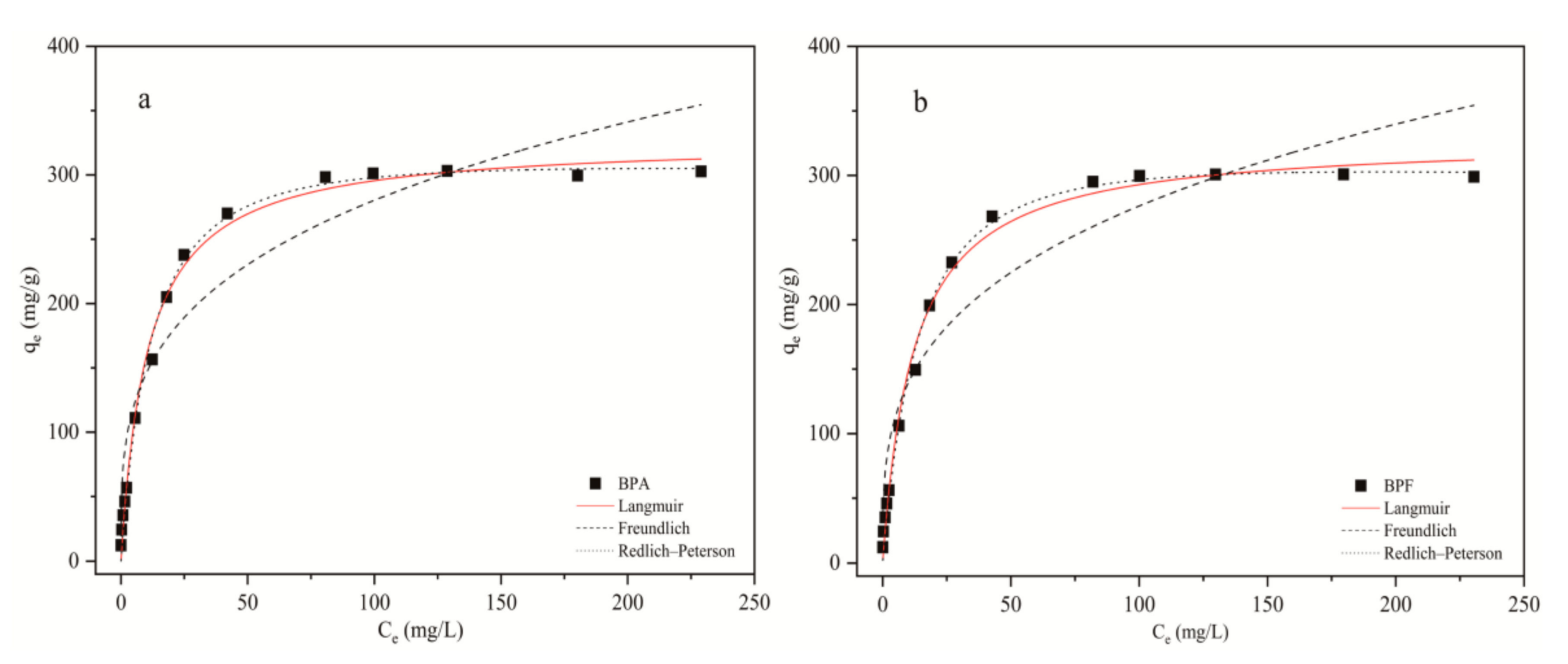
Adsorption isotherms obtained for BPA (**a**) and BPF (**b**) on MCGC.

The adsorption isotherm can intuitively reflect the relationship between the adsorption capacity of the adsorbent itself and the concentration of the adsorbate remaining in the water, which is an important parameter for understanding the adsorption process and adsorption mechanism [39]. To understand the BPA and BPF adsorption performance of the MCGC composite, its adsorption characteristics were studied under optimal adsorption conditions, and the commonly used Langmuir, Freundlich, and Redlich–Peterson isotherm models were employed to estimate the adsorption capacity and understand the adsorption mechanism. Equations (3)–(5) were used for the models [39,40,41]:

(3) $$q_{e}=\frac{q_{\max}K_{L}C_{e}}{1+K_{L}C_{e}}$$

(4) $$q_{e}=K_{F}C_{e}{}^{1/n}$$

(5) $$q_{e}=\frac{K_{RP}C_{e}}{1+\alpha_{RP}C_{e}{}^{\beta_{RP}}}$$

where *C_e_* (mg/L) is the concentration of BPA in adsorption equilibrium; *q_max_* (mg/g) and *K_L_* (L/mg) are the Langmuir model constants related to the maximum adsorption capacity and adsorption energy, respectively; and *K_F_* ((mg/g) (L/mg)1/n) and n are the Freundlich model constants related to adsorption capacity and adsorption strength, respectively. *K_F_* and 1/n are obtained from the intercept and slope of the linear curves of ln *q_e_* and ln *C_e_*, respectively. The *K_RP_* (L/mg) and *α**_RP_*** (L/mg) are the Redlich–Peterson model constants, *β_RP_* of the Redlich–Peterson constant power having values between 0 and 1. The adsorption isotherms obtained for BPA and BPF on MCGC are shown in Figure 6, and the results obtained for various parameters are shown in Table 1.

The Langmuir isotherm model is based on the assumption that the surface properties of the adsorbent are uniform, and there are few adsorption sites on the adsorbent surface. The adsorption sites are independent of each other and are not associated with whether the adjacent sites are occupied by other molecules, and adsorption occurs by single molecule layer adsorption. The Freundlich isotherm model assumes that the surface of the adsorbent is not uniform, that the adsorption energy of different adsorption sites varies, and that multilayer adsorption occurs on the surface of the adsorbent. The Redlich–Peterson isotherm model is a combination of the Langmuir model and the Freundlich model and can describe nonuniform sorption. The values of the correlation coefficient R obtained for the Freundlich, Langmuir, and Redlich–Peterson models were all greater than 0.91, but the correlation coefficient R obtained for the Redlich–Peterson model was higher, indicating that the Redlich–Peterson model may be more suitable than the Langmuir and Freundlich model for fitting the adsorption isotherms of BPA and BPF on MCGC. The results indicate that BPA and BPF exhibit multilayer adsorption behavior on the surface of MCGC and that the adsorption sites on the surface are heterogeneous. According to the Langmuir isotherm model estimation, the maximum BPA and BPF adsorption capacities for MCGC were determined to be 326.8 mg/g and 328.3 mg/g, respectively, which also demonstrates that MCGC shows relatively high BPA and BPF adsorption capacities.

### 3.4. Adsorption Kinetics

**Figure 7:**
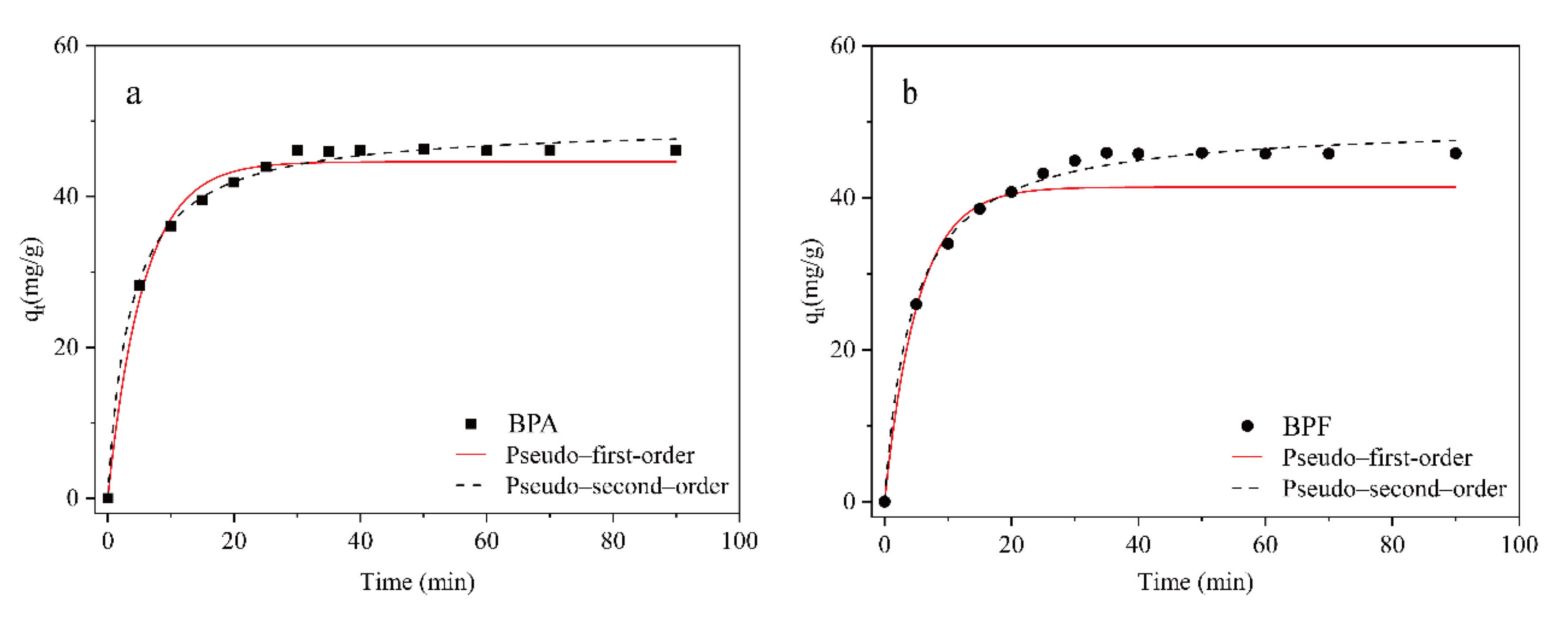
Adsorption kinetics determined for BPA (**a**) and BPF (**b**) on MCGC.

The study of adsorption kinetics is the basis for designing the adsorption process for adsorbents in large-scale practical applications and can reflect the dynamic changes in adsorption and help elucidate the adsorption mechanism [42]. To further study the dynamic changes in adsorption and the adsorption mechanism for BPA and BPF on MCGC, a pseudo-first-order kinetic model and pseudo-second-order kinetic model were used to calculate the BPA adsorption kinetics. The model equations used were as follows [43,44] are shown in Equations (6) and (7):

(6) $$q_{t}=q_{e}(1-e^{-k_{1}t})$$

(7) $$q_{t}=\frac{q_{e}{}^{2}k_{2}t}{1+k_{2}q_{e}t}$$

where *q_t_* (mg/g) is the BPA or BPF adsorption capacity of MCGC at time *t*, and *k*_1_ (min^−1^) and *k*_2_ (g/mg·min) are the rate constants for the pseudo-first-order kinetic model and pseudo-second-order kinetic model, respectively. The adsorption kinetics obtained for BPA and BPF on MCGC are shown in Figure 7, and the results obtained for each parameter are shown in Table 2.

The pseudo-first-order kinetic model assumes that there is only one binding site on the adsorbent surface, while the pseudo-second-order kinetic model assumes that there are multiple binding sites on the adsorbent surface. From Figure 7 and Table 2, it can be concluded that the correlation coefficient R values of the pseudo-first-order kinetic model and pseudo-second-order kinetic model for MCGC at the same initial concentrations of BPA and BPF were greater than 0.9, but the overall R value of the pseudo-first-order kinetic model was higher than that of the pseudo-second-order kinetic model; the *q_e_* theoretical data obtained for the pseudo-first-order kinetic model were relatively close to the experimental data. The above findings indicate that a pseudo-second-order kinetic model is more suitable for MCGC and suggest that there are multiple adsorption sites on the surface of MCGC and that BPA and BPF adsorption on MCGC mainly involve chemical adsorption.

### 3.5. Recyclability

**Figure 8:**
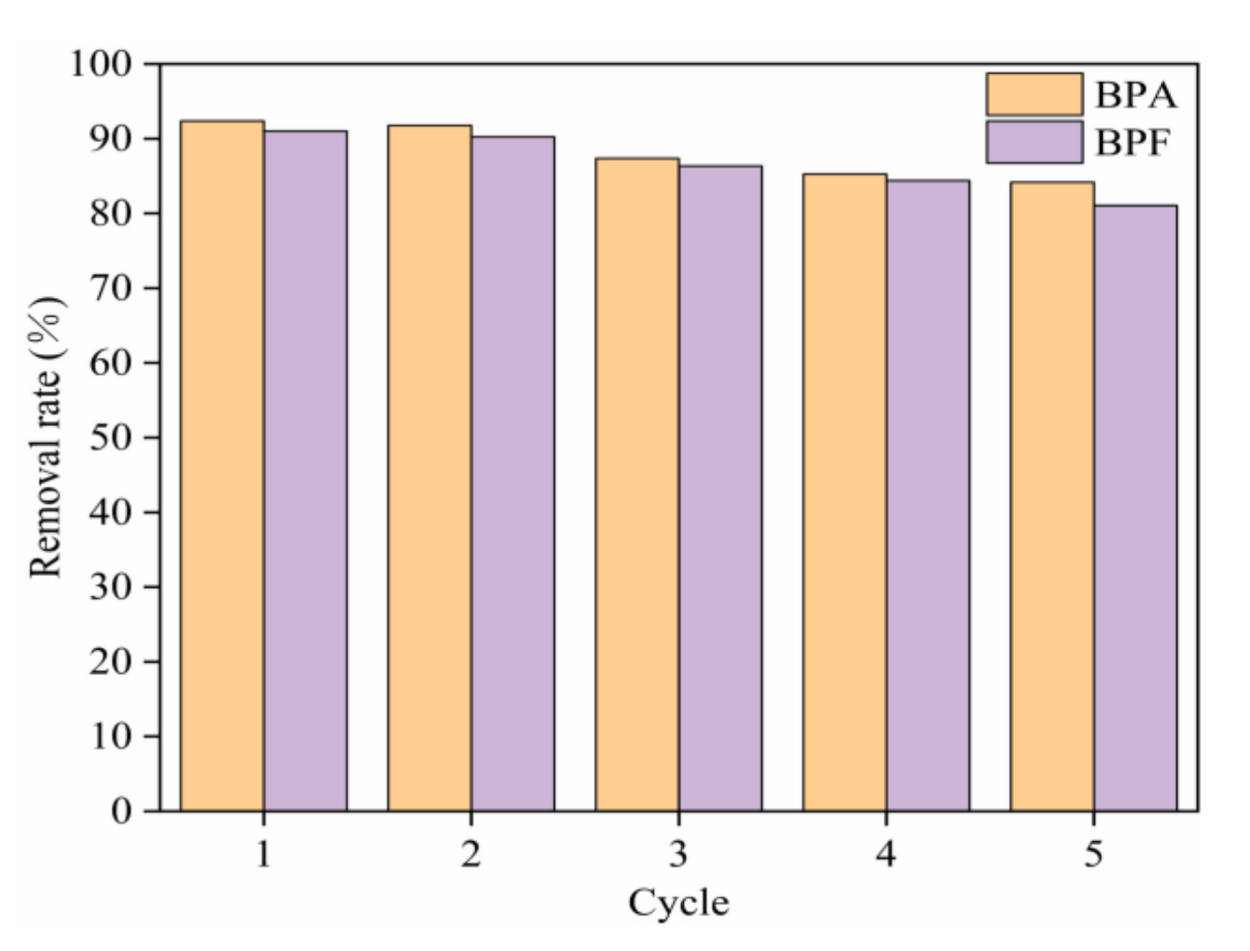
Recyclability cycles performed for MCGC.

Recyclability is an important indicator for evaluating adsorbents, and adsorbents with good recyclability are more environmentally friendly and more economical. The experiment used to remove BPA and BPF was repeated five times to evaluate the recyclability of MCGC. The results are shown in Figure 8.

After the experiment was repeated twice, almost no decrease in the BPA and BPF removal rates was found for MCGC. After the third cycle, the removal rates decreased slowly, but after the fifth cycle, the BPA and BPF removal rates were still 84.1% and 81%, respectively, showing that MCGC as an adsorbent has high stability and fine recyclability and is a green and environmentally friendly bisphenol adsorbent with certain economic value.

### 3.6. Discussion of the Adsorption Mechanism

**Figure 9:**
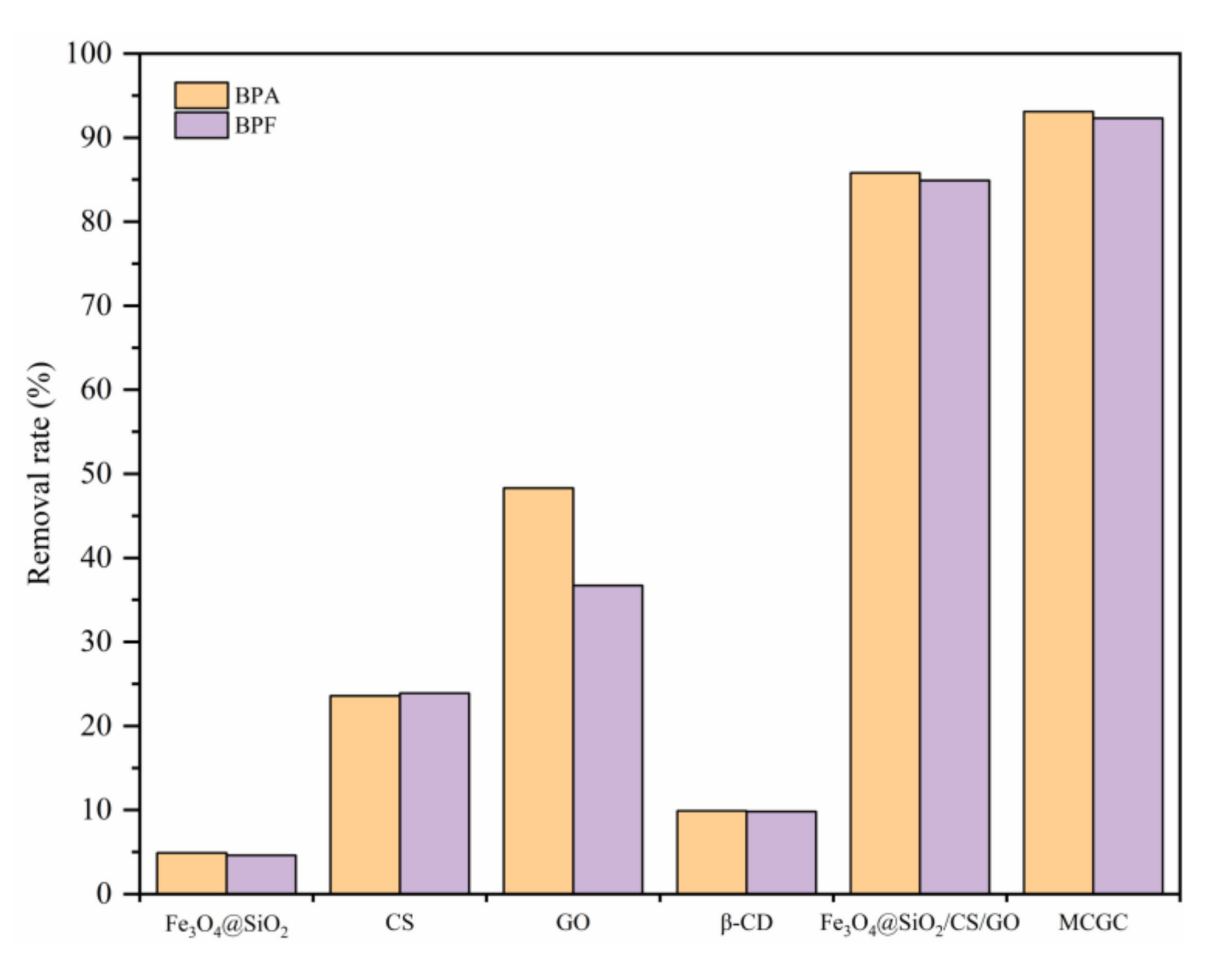
Ability of each component of the adsorbent to remove BPA and BPF in water (adsorbent amount 20 mg; adsorption time 60 min; shaking speed 200 rpm; temperature 30 ℃; initial concentration 20 mg/L; volume 50 mL; pH 7).

In order to better understand the adsorption mechanism, the removal abilities of BPA and BPF by the components and raw materials of MCGC were compared, and the results are shown in Figure 9.

The removal abilities of Fe_3_O_4_@SiO_2_ toward BPA and BPF are very low, probably because only physical adsorption occurred [25], and the ability to use the main raw materials alone such as CS, GO, and *β*-CD to remove BPF and BPF in water is far inferior to the synthesized MCGC. The results indicates that these raw materials can be converted into unique nanocomposites through compounding. In addition, the removal ability of Fe_3_O_4_@SiO_2_/CS/GO without grafted *β*-CD is approximately about 10–15% different from that of MCGC. Because both BPA and BPF are aromatic compounds that contain benzene rings; they are slightly soluble in water and highly nonpolar. The amino and hydroxyl groups on the surface of CS grafted onto MCGC may undergo electrostatic and hydrogen bonding interactions with the hydroxyl groups in the BPA and BPF molecules; the grafted GO has a benzene ring-like structure containing many oxygen-containing groups on its edges and basal plane, and may adsorb BPA and BPF through π–π attractive forces, hydrophobic forces, and hydrogen bonding interactions. Moreover, *β*-CD on the surface of MCGC has a cavity structure consisting of a hydrophobic inner cavity and a hydrophilic outer surface and may form relatively stable inclusion complexes with BPA and BPF molecules through hydrophobic forces, van der Waals forces, etc. A combination of the adsorption data and the results obtained from the adsorption isotherm model and kinetic model fitting show that the adsorption sites on the surface of MCGC are multilayer heterogeneous sites. Therefore, the adsorption mechanism for MCGC may be synergistic adsorption involving a variety of adsorption forces, and chemical adsorption is dominant with physical adsorption being a supplement.

### 3.7. Real sample verification

To evaluate the ability to treat actual wastewater, the standard addition method employed MCGC for the removal of BPA and BPF in a real sample obtained from a sewage treatment plant in Baoding (China); the real sample was stored in a brown glass bottle. Since neither BPA nor BPF was detected in real samples, after the addition of different contents of BPA and BPF (10, 30 and 50 μg/L), there are three parallels for each additive concentration, and all samples are analyzed thrice. Then, the mean method recoveries were calculated, and the results are shown in Table 3.

The results show that the recovery rates of different added BPA and BPF concentrations are above 90%; therefore, MCGC has certain application value.

### 3.8. Comparison with Other Adsorbents

The adsorption capacity of MCGC was compared with that found in the past for other adsorbents used to adsorb BPA and BPF. The results are shown in Table 4.

It can be seen from Table 4 that although the adsorption capacity (removal) of MCGC for BPA and BPF is not the highest, it is relatively impressive. This is due to the multiple adsorption forces of MCGC for synergistically adsorbing BPA and BPF. The results indicate that MCGC is an efficient BPA and BPF adsorbent and can be used as a magnetic adsorbent to remove phenolic pollutants in water.

## 4. Conclusions

In this study, a new type of high-efficiency adsorbent, MCGC, was successfully prepared by using environmentally friendly and economical raw materials and multi-step methods, and an attempt to simultaneously remove BPA and BPF from water using this material was carried out. Fe_3_O_4_ was used as a magnetic core to facilitate solid–liquid separation and adsorbent collection, and a SiO_2_ coating was used to enhance the stability and dispersibility of the magnetic particles. Subsequently, CS, which contains numerous amino groups that can be grafted by amidation with the carboxyl group on the surface of GO, was crosslinked onto the surface of Fe_3_O_4_@SiO_2_. After grafting, the composite material and β-CD formed an adsorbent with a synergistic effect for multiple adsorption mechanisms through hydrogen bonding interaction [31,32]. MCGC was characterized by SEM, TEM, FTIR, XRD, and MPMS. The results showed that the morphology and crystal structure of MCGC were good, its stability was high, and the material could be separated quickly by an external magnetic field. An adsorption optimization study showed that the BPA and BPF removal rates increased with increasing adsorbent dosage, shaking speed, and time. Neutral pH, no ionic strength, and low temperature (30 °C) were found to be beneficial for realizing the maximum adsorption capacity. Through fitting and calculation using an adsorption isotherm model and kinetic model and exploration of the adsorption mechanism, the results obtained showed that the adsorption process for BPA and BPF on MCGC might involve multilayer adsorption and synergistic adsorption based on multiple forces, including π–π attractive forces, hydrophobic forces, hydrogen bonding interactions, and van der Waals force. The maximum BPA and BPF adsorption capacities were determined to be 326.8 mg/g and 328.3 mg/g, respectively. After five cycles of adsorption, MCGC still showed removal rates of 84.1% and 81% for BPA and BPF, respectively. And through real sample verification, MCGC has certain application value. In addition, the raw materials used to prepare the adsorbent are inexpensive and easy to obtain. However, the adsorbent has certain application prospect, because its recycling is relatively time-consuming, which may hinder the effective application of the adsorbent in industry; therefore, the possibility of using these extremely valuable composites to decorate substrates, such as sponges, aerogels and other monoliths, for the fabrication of reusable devices should be the focus of future research. In summary, MCGC is a promising high-efficiency adsorbent that can also be promoted and applied to adsorb other environmental pollutants.

## Figures and Tables

**Table 1:** Adsorption isotherm parameters obtained for bisphenol A (BPA) and bisphenol F (BPF) on Fe_3_O_4_@SiO_2_/chitosan/graphene oxide/β-cyclodextrin (MCGC).

Adsorbate	Langmuir			Freundlich			Redlich–Peterson			
	*q_max_* (mg/g)	*K_L_*	*R*	*1/n*	*K_F_*	*R*	*K_RP_*	*α_RP_*	*β_RP_*	*R*
BPA	326.8	0.094	0.992	0.283	75.9	0.914	26.46	0.06	0.95	0.993
BPF	328.3	0.083	0.992	0.298	70.1	0.911	22.36	0.05	0.97	0.994

**Table 2:** The kinetic parameters obtained for BPA and BPF adsorption on MCGC.

Adsorbate	Pseudo-First-Order			Pseudo-Second-Order		
	*q_e_* (mg/g)	*k*_1_	*R*	*q_e_* (mg/g)	*k*_2_	*R*
BPA	44.6	0.18	0.986	46.5	0.01	0.994
BPF	41.3	0.19	0.932	45.8	0.01	0.995

**Table 3:** Results of the adsorption of BPA and BPF in real wastewater samples under the optimum conditions (*n* = 3).

Adsorbate	Added Concentration (μg/L)	Recovery Rate (%)
BPA	10	91.3
	30	97.1
	50	95.8
BPF	10	90.2
	30	94.6
	50	97.7

**Table 4:** Comparison of the adsorption capacities of various adsorbents for BPA and BPF removal.

Adsorbates	Adsorbents	Adsorption Capacity (mg/g)	Removal (%)	References
BPF	DMIP	18.6		[45]
BPA	SB-beta-CD	121		[46]
BPA	Biochar KLP	220		[47]
BPF	N-GLCS	222.9		[48]
BPA, BPF	Fe_3_O_4_@Co/Ni-LDH	238.96, 177.09		[49]
Phenol	PCL45/GO		100	[50]
Phenol	PCL80/GO		>95	[50]
BPA	TiO_2_-rGH	476.2	100	[51]
BPA	MIL-53(Al)	325		[52]
BPA	MIL-53(Al)-F127	465		[52]
BPA, BPF	MCGC	326.8, 328.3	93.1, 92.3	This work
